# A highly effective and versatile technology for the isolation of RNAs from grapevines and other woody perennials for use in virus diagnostics

**DOI:** 10.1186/s12985-015-0376-3

**Published:** 2015-10-20

**Authors:** Huogen Xiao, Won-Sik Kim, Baozhong Meng

**Affiliations:** Department of Molecular and Cellular Biology, University of Guelph, Guelph, 50 Stone Road East, Guelph, ON N1G 2W1 Canada; Norgen BioTek, Thorald, ON L2V 4Y6 Canada

**Keywords:** RNA isolation, Woody plants, Grapevines, Polyvinylpyrrolidone, Viral diagnosis, RT-PCR, RT-qPCR, Small RNA and microRNA

## Abstract

**Background:**

Isolation of pure RNA from woody perennials, especially fruit crops such as grapevine rich in complex secondary metabolites, has remained very challenging. Lack of effective RNA isolation technology has resulted in difficulties in viral diagnosis and discovery as well as studies on many biological processes of these highly important woody plants. It is imperative to develop and refine methodologies with which large amounts of pure nucleic acids can be readily isolated from woody perennials.

**Methods:**

We compared five commonly used RNA isolation kits in isolating total RNA from twelve species of woody perennials. We made modifications to select RNA isolation systems to simplify and improve their efficiency in RNA isolation. The yield and quality of isolated RNAs were assessed via gel electrophoresis and spectrophotometric measurement. We also performed RT-PCR and RT-qPCR to detect several major viruses from grapevines.

**Results:**

Two of the kits were shown to be the best in both the yield and quality of the isolated RNA from all twelve woody species. Using disposable extraction bags for tissue homogenization not only improved the yield without affecting quality, but also made the RNA isolation technology simpler, less costly, and suitable for adoption by many potential users with facility limitations. This system was successfully applied to a wide range of woody plants, including fruit crops, ornamentals and timber trees. Inclusion of polyvinylpyrrolidone in the extraction buffer drastically improved the performance of the system in isolating total RNA from old grapevine leaves collected later in the season. This modification made our system highly effective in isolating quality RNA from grapevine leaves throughout the entire growing season. We further demonstrated that the resulting nucleic acid preparations are suitable for detection of several major grapevine viruses with RNA or DNA genomes using PCR, RT-PCR and qPCR as well as for assays on plant microRNAs.

**Conclusions:**

This improved RNA isolation system would have wide applications in viral diagnostics and discovery, studies on gene expression and regulation, transcriptomics, and small RNA biology in grapevines. We believe this system will also be useful in diverse applications pertaining to research on many other woody perennials and recalcitrant plant species.

**Electronic supplementary material:**

The online version of this article (doi:10.1186/s12985-015-0376-3) contains supplementary material, which is available to authorized users.

## Background

Woody plants, such as timber trees, fruit trees, and ornamental trees, are of great importance to people, not only economically and ecologically but also bio-aesthetically [1]. Woody fruit crops are generally infected with a multitude of taxonomically diverse viruses, which not only affect fruit quality and yield, but also cause degeneration and loss of vitality, stock-scion incompatibilities and tree death. For instance, stone fruits are infected with 36 viruses from 11 genera and six families [2]. Grapevines are infected by 64 distinct species of viruses belonging to 27 genera and 15 families [3, 4]. Moreover, new viruses continue to be identified in woody fruit crops, due to the advent and application of highly effective RT-PCR and next generation sequencing [5–9]. Although little information is available on plant viruses of forest trees, infections with viruses may contribute to the decline of forest [10]. Explorations of plant viruses in forest ecological systems would allow the discovery of novel viruses, which would be of great importance for the study of origin, evolution as well as the ecology of plant viruses.

For the control of viral diseases in woody fruit crops, the most important strategy is prevention through the use of virus-free propagation materials during orchard and vineyard establishment. Development and implementation of rapid, highly sensitive and reliable diagnostic methods is the key to plant health in woody fruit crops and constitutes an integral component of the virus-free certification programs in many countries. Currently, the official methods for diagnosis of woody fruit crop viruses are largely biological indicator indexing and serological methods [11]. These methods suffer from insufficient sensitivity and specificity, lengthy procedure, and high cost due to high demand for labor. Nucleic acid-based diagnostics, such as RT-PCR and qRT-PCR, are advantageous over biological and serology-based methods for viral diagnosis due to their superb sensitivity, specificity, reliability and speed [12].

As one of the most powerful technologies ever developed in recent years, deep sequencing makes possible the global identification of virtually all viruses and viroids present in an infected plant, which would otherwise be impossible by conventional, sequence-specific RT-PCR-based assays [13]. Deep sequencing can be performed using a variety of nucleic acid preparations, which include total RNAs [14], double-stranded RNAs [5, 6] and small RNAs [7, 8, 15]. Since deep sequencing can retrieve massive amounts of sequence data, unknown viruses that may contribute to the development of a complex disease in an infected plant can be unveiled. This technology has been used by several research groups for diagnosis of viruses and for discovering novel viruses [4, 5, 7–9, 14–18], which, in turn, will facilitate understanding of the complicated etiology, molecular biology of the viruses involved in complex diseases, and virus-host interactions.

The success of deep sequencing, RT-PCR and RT-qPCR is contingent upon quality RNAs. Unfortunately, isolation of highly pure RNA from woody plants has been very challenging and problematic compared to herbaceous plant species. This is due to the presence of high levels of secondary metabolites, such as polyphenols and polysaccharides, in woody plants. Polyphenolic substances react with RNAs and form insoluble complexes, thus severely interfering with RNA isolation, resulting in low yield and poor quality RNAs. Consequently, the isolated RNAs are not suitable for downstream applications such as RT-PCR, RT-qPCR and deep sequencing. These issues were circumvented through the use of double-stranded RNAs to serve as template for viral genome sequencing and PCR-based diagnosis [19–25]. However, the procedure for isolation of dsRNA is lengthy, limited to small sample size and involves the use of hazardous organic solvents such as phenol and chloroform. To resolve these issues, researchers have attempted numerous methods to isolate total RNA. But these protocols still take days to carry out, require extraction with hazardous organic solvents, and involve many steps which could result in problems with cross contamination in downstream virus detection. For example, Tattersall et al. [26] compared 15 methods for isolating RNAs from grape leaves and concluded that the Tris-LiCl method was most effective. However, this procedure is highly time-consuming, requires a span of 5 days and a total of 33 steps to accomplish, posing a major limitation for its wide use for diagnostic purposes. A procedure based on homogenization in an extraction buffer containing cetyltrimethylammonium bromide (CTAB) and selective precipitation of RNA with LiCl requires two days for isolation of RNA from peach or other woody plants [27–30]. An improved procedure, the rapid CTAB method, still requires three hours to complete [31].

A number of RNA isolation kits have been developed by biotech companies and used for isolation of RNAs from diverse plant species, mostly annual, herbaceous plants [32–35]. These kits all use a silica-based column, involves a simple and straightforward procedure, avoid the use of organic solvents and yield quality total RNA within a short period of time. Some of the kits have been attempted for isolating RNA from woody plants with various degrees of success and inherent pitfalls [16, 36–39]. However, a systematic comparison of the effectiveness of these commercial kits in isolating nucleic acids from grapevine tissues and other woody plants has not been reported in the literature.

The objectives of this study were to compare and refine RNA isolation methodologies that are commercially available for use in isolation of sufficient quantities of quality RNAs from a wide range of woody plant species. We have identified the best commercial kits for the isolation of RNAs (including both large and small RNAs). We have further simplified the RNA isolation technology so that they could be adopted by a large spectrum of users. Finally, we have also made significant improvement to the RNA isolation methodology, so that it is suitable for the detection of both RNA and DNA viruses using grapevine tissues collected throughout the growing season.

## Results

### Comparison of five commercial kits in isolating RNA from peach and grapevine

Five commonly used commercial RNA isolation kits were compared for their effectiveness in isolation of RNA from woody plants. These five kits are TRIzol Reagent (Life Technologies), RNeasy Plant mini kit (Qiagen), Spectrum™ Plant Total RNA kit (Sigma), AccuPrep viral RNA extraction kit (Bioneer) and Plant/fungi total RNA kit (Norgen BioTek). These kits were chosen due to their advantages in certain aspects in RNA isolation. RNeasy plant mini kit has been used for a long time for isolating RNA or cleaning up crude RNA preps isolated using various methods from different plant species, including woody plants [32, 34, 37]. The Spectrum™ Plant Total RNA kit (Sigma) has been recently reported for the isolation of quality RNA from woody plants [14, 16, 36, 40, 41]. The Plant/fungi total RNA kit (Norgen BioTek) has been used to isolate total RNA from plants and fungal pathogens and is especially effective for small RNA [35, 38, 39]. The AccuPrep viral RNA extraction kit (Bioneer) has been reported in isolating viral RNA [42]. Lastly, the TRIzol Reagent was chosen as a conventional method based upon a guanidinium thiocyanate-phenol-chloroform extraction method originally described by Chomczynski and Sacchi [43], which has been widely used to isolate both large and small size RNAs, DNA and proteins from a wide range of sources, including many plant species [44].

**Fig. 1:**
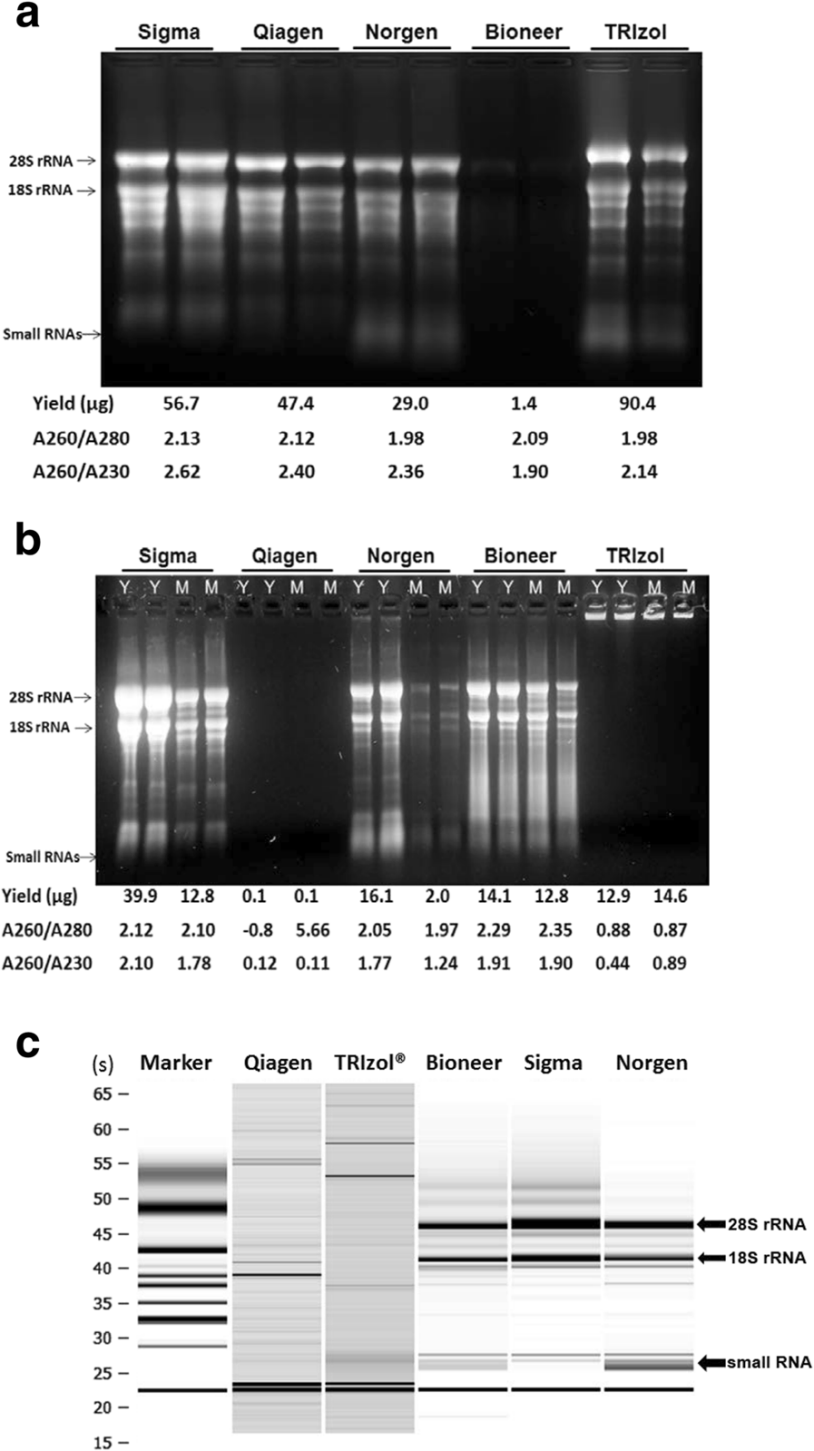
Profile of total RNA isolated by using five commercial kits. **a** Denaturing gel electrophoresis of total RNA isolated from peach. **b** Denaturing gel electrophoresis of total RNA from grapevine leaves. 50 mg of young peach and grapevine leaves (indicated as Y), and mature (M) grapevine leaves was used in RNA isolation with Spectrum™ Plant Total RNA kit (Sigma), RNeasy Plant mini kit (Qiagen), Plant/fungi total RNA kit (Norgen), AccuPrep viral RNA extraction kit (Bioneer) and TRIzol Reagent (Life Technologies). The total RNA yield (μg), A260/A280 and A260/A230 ratios averaged from two replicates are given below each gel panel. 28S rRNA, 18S rRNA and small RNAs are indicated with arrows. **c** Capillary electrophoresis of total RNA with Agilent Bioanalyzer. One ml of each of the total RNA preparations isolated using these five systems was used for the analysis with an Agilent Bioanalyzer 2100 equipped with an RNA Nano chip

The five kits were first compared for their performance in isolating total RNA from young peach leaves. The RNA integrity was assessed by visualization of ribosomal RNA bands on 1.5 % MOPS/formaldehyde gel. As shown in Fig. 1a, all the five kits produced high quality RNAs as judged by distinct 28S and 18S rRNA bands without signs of degradation. The quantity and quality of extracted RNA was also estimated using spectrophotometer analysis (Fig. 1a). For all samples the A260/A280 ratios were 1.98 or higher and the A260/A230 ratios were 1.90 or higher, indicating that the RNAs were of high purity. However, the yields of total RNA extracted using different kits varied considerably. TRIzol method produced the highest yield of total RNA, with 90.4 μg per isolation from 50 mg of leaf tissue, followed by Sigma (56.7 μg), Qiagen (47.4 μg) and Norgen’s kit (29.0 μg). Bioneer’s kit produced the lowest yield at only 1.4 μg. For the small RNAs, both TRIzol and Norgen’s kit produced higher yield than the other three kits based on intensity of low molecular weight RNA bands on the gel (Fig. 1a).

We then used these five kits to isolate total RNA from both young and fully expanded leaves of grapevine (*V. vinifera* cv. Chardonnay). The results showed that the Spectrum plant total RNA kit from Sigma gave the best RNA yield of 39.9 μg per isolation from young leaves, followed by kits from Norgen and Bioneer (Fig. 1b). The Sigma, Norgen and Bioneer kits all produced high quality RNAs as indicated with an over 2.0 of A260/A280 (Fig. 1b) and with a RIN of 9.0, 8.9 and 7.7, respectively (Fig. 1c and data not shown). Regardless of either young or fully expanded leaves were used, Qiagen’s kit failed to isolate RNA using either the RLT or the RLC lysis buffers as judged by the electrophoretic profile on denaturing agarose gels (Fig. 1b and data not shown). Similarly, Trizol® reagent also failed to isolate RNA from grape leaves due to the insolubility of the pellet containing RNA (Fig. 1b).

As expected, the Sigma, Norgen and Bioneer kits all produced less RNA from fully expanded leaves compared to young leaves (Fig. 1b). It was also shown that Norgen’s kit produced more of low molecular weight RNAs than the Sigma and Bioneer kits from both young and mature leaves of grapevine (Fig. 1b). To confirm these observations, Agilent Bioanalyzer analysis and miRNA RT-qPCR were used to further quantify the amount of small RNAs produced from young grapevine leaves by the five kits and the results are described later.

### Application and further confirmation of the optimized RNA isolation protocol for the detection of grape viruses by RT-PCR and RT-qPCR

**Fig. 2:**
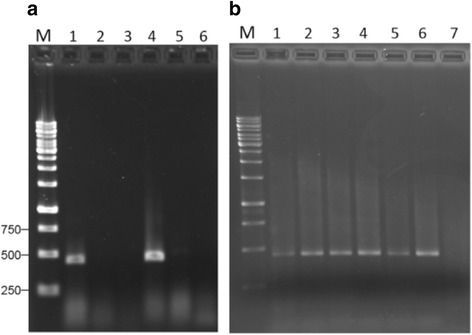
RT-PCR detection of GRSPaV from RNAs isolated with Spectrum™ Plant Total RNA kit (Sigma). **a** Agarose gel analysis of RT-PCR products with primers RSP21 and RSP22 targeting the capsid protein gene (Additional file 1: Table S1) on total RNA extracts from *V. vinifera* var. Chardonnay (lane 1), *V. riparia* from Quebec (lane 2), *V. riparia* from Manitoba (lane 3), *V. vinifera* var. Riesling (lane 4) and *V. vinifera* var. Thompson seedless (lane 5). Lane M: molecular size marker (bp); lane 6: water control. **b** Agarose gel analysis of RT-PCR products using total RNA extracts from *V. vinifera* var. Syrah. Lanes 1–6: leaf samples from six individual vines. Lane 7: water. Lane M: molecular size marker (bp)

To test the quality of the RNAs isolated with the commercial kits, RT-PCR and RT-qPCR were carried out to detect virus in grapevine plants. The total RNA used for RT-PCR detection were isolated with Sigma’s kit from leaf samples collected from grapevine plants growing in a growth chamber. Plants tested included commercial varieties of *V. vinifera*: Chardonnay, Riesling and Thompson seedless, and wild grape *V. riparia* originating from Quebec and Manitoba. In addition, leaves were also collected from *V. vinifera* var. Syrah from a commercial vineyard. cDNAs were made from these RNA samples using High-capacity cDNA Reverse Transcription kit (Life Technologies). Subsequent PCR amplification was carried out with primers RSP21 and RSP22 targeting the capsid protein gene of *Grapevine rupestris stem pitting-associated virus* (GRSPaV) (Additional file 1: Table S1). The expected size of the amplified products is 441 bp and the specificity of amplification was confirmed by cloning and sequencing the RT-PCR amplicons (data not shown). GRSPaV was detected from *V. vinifera* var. Chardonnay (Fig. 2a, lane 1) and *V. vinifera* var. Riesling (Fig. 2a, lane 4). Interestingly, it was not detected from wild grape *V. riparia* collected either from Quebec (Fig. 2a, lane 2) or Manitoba (Fig. 2a, lane 3). GRSPaV was detected from all the six Syrah samples collected from a commercial vineyard (Fig. 2b).

**Fig. 3:**
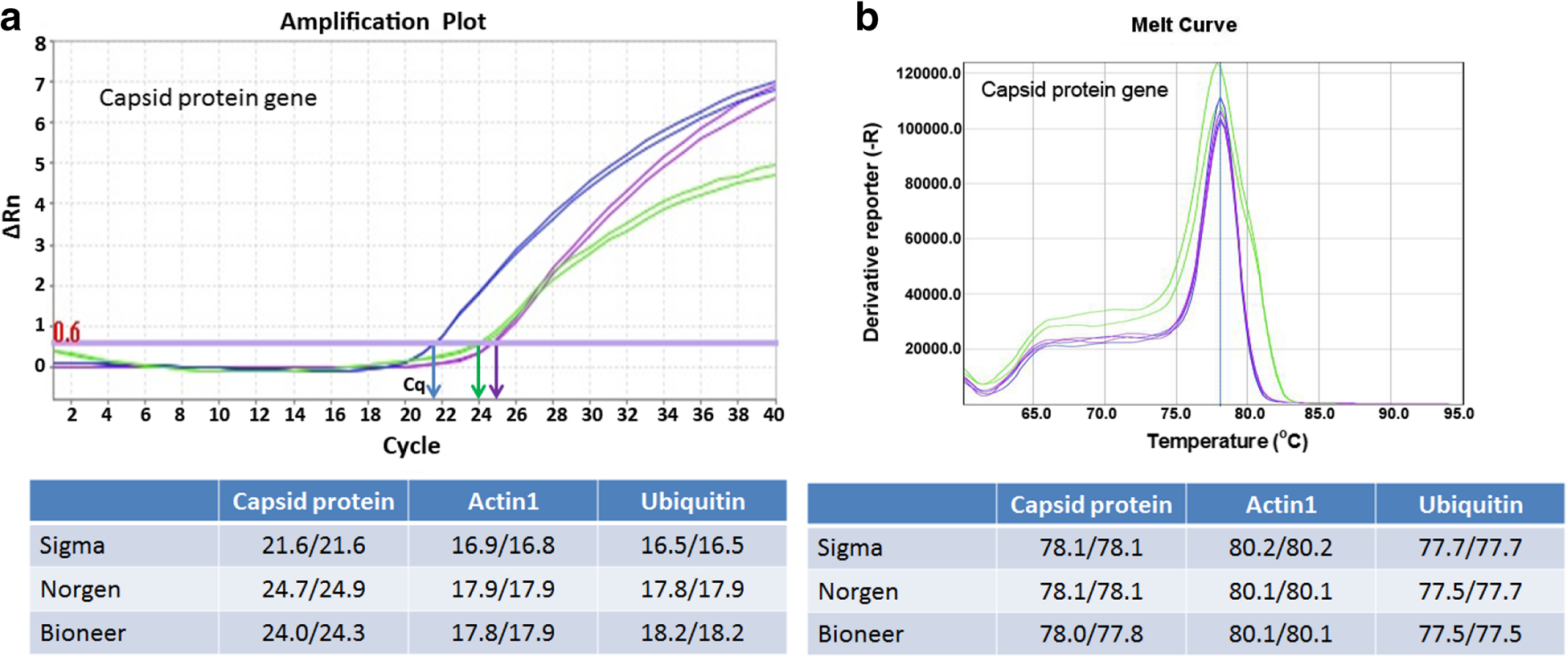
RT-qPCR detection of GRSPaV from total RNA isolated from grapevine leaves. Total RNA was isolated from *V. vinifera* var. Chardonnay using Spectrum™ Plant Total RNA kit (Sigma), Plant/fungi total RNA kit (Norgen) and AccuPrep viral RNA extraction kit (Bioneer). cDNA prepared with oligo d(T) on 2 μg of total RNA were subjected to SYBR Green quantitative real-time PCR with primers targeting GRSPaV capsid protein gene, grape actin1 and the gene encoding ubiquitin-60S ribosomal protein L40-2 (Additional file 1: Table S1). Shown is the amplification plot (**a**) and melt curve (**b**) from cDNAs prepared from RNAs isolated with Sigma (blue), Norgen (purple) and Bioneer (green). The C_q_ and melting temperature (T_m_) of two technical replicates for all the samples and genes are given in the table below

RNAs isolated from Chardonnay leaves with Sigma, Norgen and Bioneer RNA kits were used for two steps RT-qPCR detection. Internal controls were included by amplifying grape genes encoding actin 1 and ubiquitin-60S ribosomal protein L40-2 with primers designed to span exon-exon junction (Additional file 1: Table S1). SYBR Green dye was used in RT-qPCR due to its lower cost and versatility. The results of RT-qPCR with primers RSP8277F and RSP8436R targeting the capsid protein (CP) gene of GRSPaV (Additional file 1: Table S1) confirmed that the RNAs isolated with Sigma, Norgen and Bioneer kits are qualitatively and quantitatively satisfactory in the detection of GRSPaV in grapevine as indicated with low quantitation cycle (Cq) values and single, tight peaks on the melt curves (Fig. 3). The results (Fig. 3a) also showed that the Cq values of total RNA obtained from Sigma kit were 3.2 and 2.25 cycles lower (for CP gene) than those from Norgen and Bioneer kits, respectively; and that the Cq values of RT-qPCR using total RNA from Sigma kit for actin 1 were 1.05 and 1 cycles lower than those from Norgen and Bioneer kits, respectively. Similar results were obtained for ubiquitin. This suggests that the Sigma kit produced better quality RNA with less inhibitors than those of Norgen and Bioneer. In summary, the RNA preparations from young and mature leaves of both growth chamber and field-grown grapevine were successfully used in downstream applications such as RT-PCR and real-time PCR.

### Evaluation of small RNAs isolated with the commercial kits

As the above results (Fig. 1) show, the Norgen kit seemed to produce more small RNAs than the other kits. To confirm this observation, Agilent Bioanalyzer analysis and miRNA RT-qPCR were conducted to further quantify the amount of small RNAs and miRNAs produced from young grapevine leaves by the five kits. The data from Agilent Bioanalyzer (Additional file 1: Table S2) clearly show that Norgen kit produced the highest yield of small RNA (4,150 ng) and miRNA (143 ng), followed by the Bioneer system. The Sigma system produced the lowest yield of small RNA (10 ng), although it produced the highest yield of total RNA. The results from RT-qPCR for miR 156a and miR 159a support that Norgen’s kit generated the highest yield of miRNA, followed by Bioneer’s kit. These results demonstrated that the Norgen kit is capable of isolating both high and low molecular weight RNAs from woody plants. In line with this, the Norgen kit has been used to isolate total RNA for the profiling of small RNAs in avocado and citrus plants [38, 39].

### Further simplification and improvement on RNA isolation methodologies

All the five kits required the use of liquid nitrogen, and mortars and pestles to grind the leaf tissues into fine powder before further processing for RNA isolation. This requirement poses a major limitation to the wide application of these technologies, as liquid nitrogen may not be available to many users and mortars and pestles would need to be washed and baked for the inactivation of RNases each time they are to be used. We, therefore, investigate the use of BIOREBA extraction bags in the homogenization and lysis of grape tissues for RNA isolation without the need of liquid nitrogen, and mortars and pestles.

**Fig. 4:**
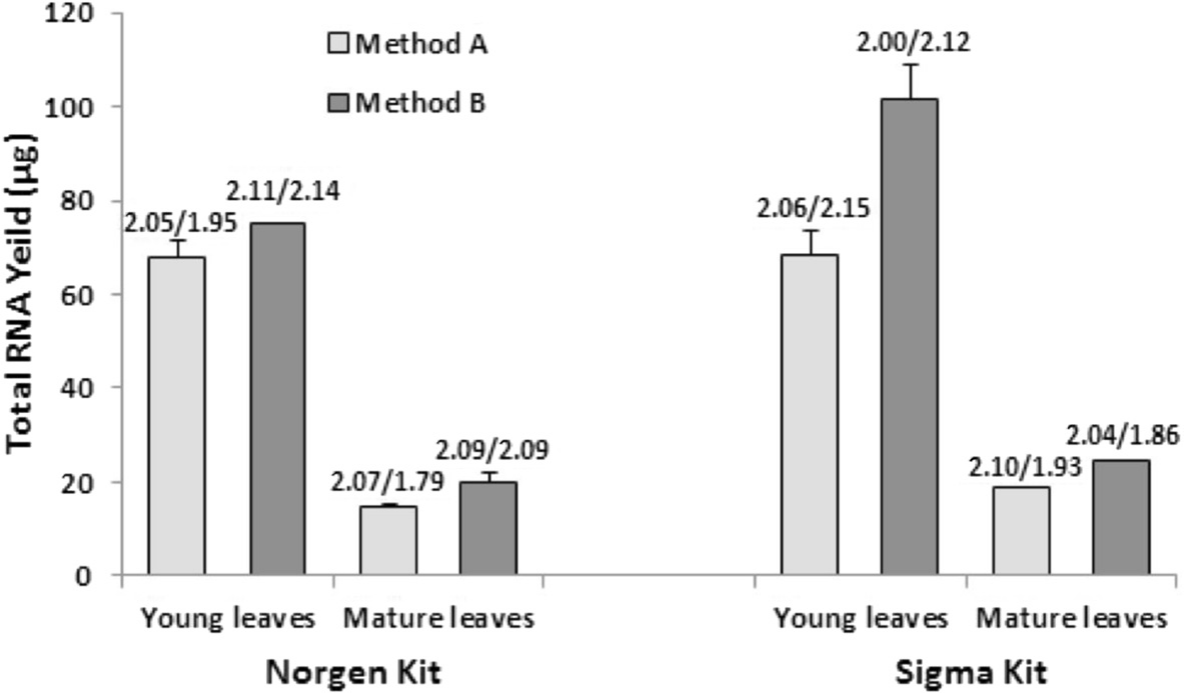
Effects of tissue homogenization and extraction methods on the RNA isolation from grapevine leaves. Young and mature leaves were homogenized by grinding in liquid nitrogen with mortar and pestle (Method A), or in BIOREBA extraction bag with lysis buffer (Method B), and then used to isolate total RNA with Plant/fungi total RNA kit (Norgen) and Spectrum™ Plant Total RNA kit (Sigma). Shown are the averages of total RNA yields of two replicates and their standard deviation. A260/A280 (on the left) and A260/A230 (on the right) ratios averaged from two replicates are given on the top of RNA yield bar

In Method A (the default method), young and mature grapevine leaves were ground in liquid nitrogen to fine powder using a mortar and pestle and a portion of the resulting fine powder (50 mg) was used in RNA isolation without further grinding in the extraction buffer. For method B, 100 mg of leaf tissue was ground in an extraction bag containing 1 ml of lysis solution with a hand-held homogenizer until the tissue was completely macerated. We used 100 mg of leaf tissue in this method because only half of the resulting lysate could be collected after grinding. The collected lysate, of 500 μl in volume, was then processed using kits from either Sigma or Norgen. As shown in Fig. 4, homogenization of grape leaves by Method B produced 10-50 % higher yield of RNA as compared to Method A, the default method recommended in both the Sigma and the Norgen systems, for both young and mature leaf tissues. We concluded that neither liquid nitrogen nor mortar and pestle is necessary in the RNA isolation from grape leaves. As a matter of fact, the method of using BIOREBA bags produced significantly more RNA with similar quality (Fig. 4). Furthermore, it allowed for processing of samples with reduced time and costs, making it suitable for high-throughput assays. The reason that homogenization of grape leaves in BIOREBA bags increased the RNA yield may have been due to the fact that the tissue has been ground more thoroughly so that more RNA could be released into lysis solution. To test this, we have tried to homogenize young grape leaves in lysis buffer with mortar and pestle without liquid nitrogen (Method C) and it produced 67 % higher yield of total RNA than that of the method A (data not shown). Kalinowska et al. [48] also reported that further grinding plant tissues in extraction buffer after the initial grinding in liquid nitrogen greatly increased the RNA yield.

### Potential applications of the improved RNA isolation methods for a wide range of woody plants

**Fig. 5:**
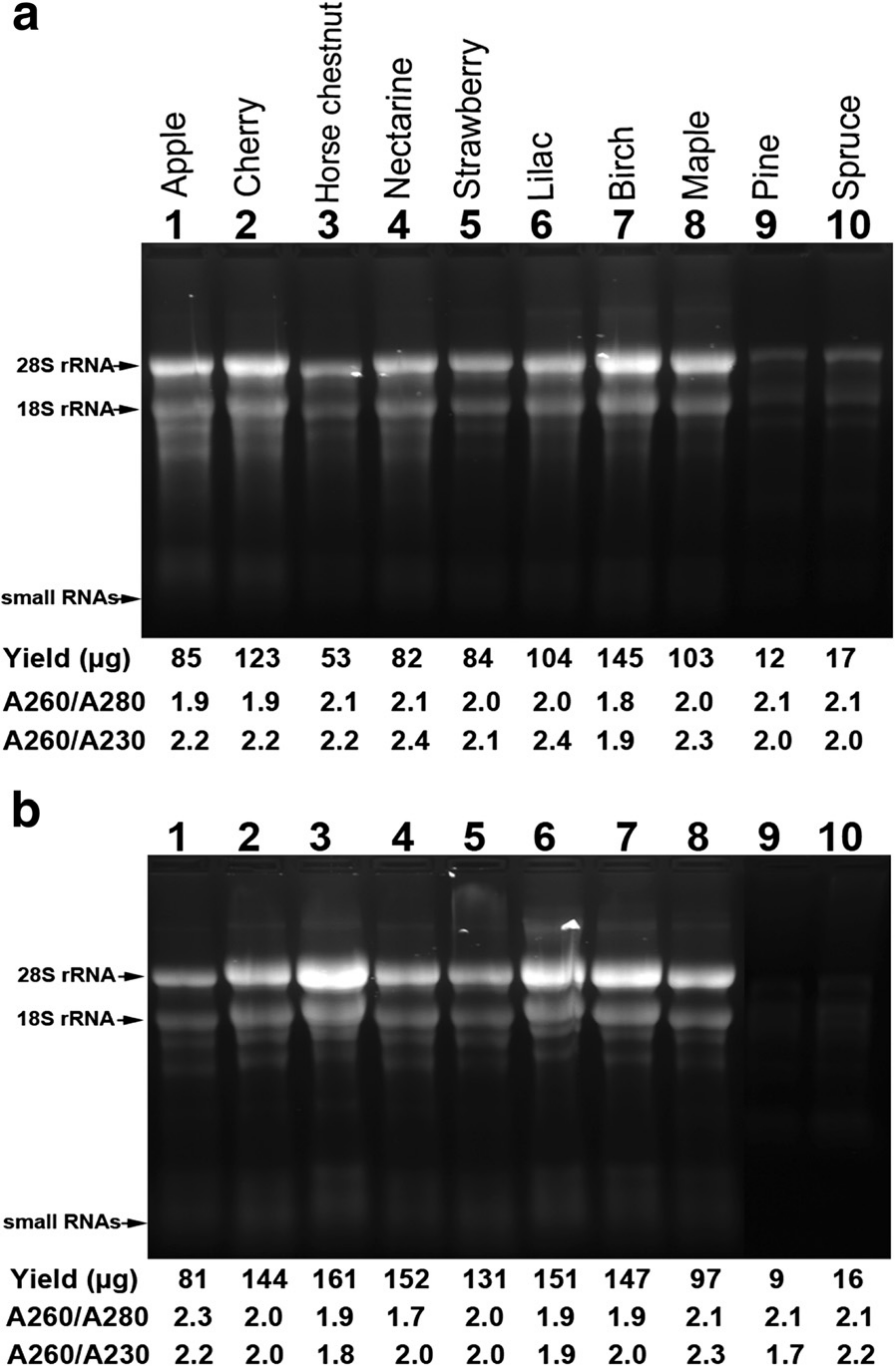
Denaturing gel electrophoresis of total RNA isolated from different woody plants and strawberry using Spectrum™ Plant Total RNA kit (Sigma) (**a**) and Plant/fungi total RNA kit (Norgen BioTek) (**b**). 100 mg of young leaves from each plant as indicated were used in RNA isolation. Homogenization of leaf tissues was done using an extraction bag from BIOREBA. This experiment was conducted twice and consistent results were obtained. Only one set of samples is shown here. 7.5 μl of RNA was loaded in 1.2 % of agarose gel. The total RNA yield (μg), A260/A280 and A260/A230 ratios are given below each gel panel. 28S rRNA, 18S rRNA and small RNAs are indicated with arrows

We then investigated the suitability of the improved procedure for RNA isolation from a wide range of woody plants. The woody plants tested were trees - paper birch, Norway maple, white pine and white spruce, fruit trees – cherry, nectarine, and apple, and ornamentals – lilac and horse chestnut. In addition, strawberry, an important herbaceous perennial species that is difficult with isolation of quality RNA [49], was also tested. The results showed that both Sigma kit (Fig. 5a) and Norgen kit (Fig. 5b) were effective in RNA isolation for all these wood plants, with high quality as judged by high A260/A280 and A260/A230 ratios (both at around 2.0), and with high yields (53–145 μg) for angiosperms (apple, cherry, horse chestnut, nectarine, lilac, birch and maple), while much lower yield (9–17 μg) for gymnosperms (spruce and pine). The yields of RNA isolated from gymnosperms are usually low [30, 50].

### Inclusion of polyvinylpyrrolidone (PVP) in the lysis buffer is critical for isolating RNA from old grapevine leaves

We then tested the effectiveness of the established methodology in isolation of total RNA from red-fruited cultivars of wine grapes (Cabernet Franc) collected later in the season showing leafroll disease symptoms. To our surprise the RNA isolation methodology was not effective at all for isolating RNAs from symptomatic leaves. As shown in Table 1, the standard Sigma kit (Standard) produced total RNA with very low yield (only 2.4 μg on average) and very low quality as indicated by an average of 1.03 for A260/A280 and 0.32 for A260/A230. As expected, GRSPaV and the reference gene ubiquitin could not be amplified from these RNA samples with RT-PCR (Additional file 2: Figure S1A and S1C). The total RNA isolated with Norgen kit from these samples were also not usable (data not shown). We made further efforts to tackle this problem and found that modification of the Sigma system by inclusion of 2.5 % PVP-40 in the lysis buffer (Modified) dramatically improved its performance in isolating RNA from old and symptomatic leaves collected in late summer and the fall. The modified method increased the yield of total RNA to 10.0 μg on average, with high quality as judged by an average of 2.04 for A260/A280 and 1.96 for A260/A230. RT-PCR results showed that GRSPaV and the ubiquitin gene were successfully detected from all these RNA samples (Additional file 2: Figure S1B and S1D). In contrast, addition of 2.5 % PVP-40 in the lysis buffer of Norgen kit, however, did not improve its performance (data not shown).

**Table 1:** Total RNA isolated from old leaves of grapevines with Sigma kit using the standard or modified protocol

Leaf samples	RNA yield (μg)		A260/280		A260/230	
	Standard	Modified	Standard	Modified	Standard	Modified
CF-1	3.1	8.8	1.08	2.08	0.29	2.00
CF-2	1.2	8.5	1.49	2.06	0.48	2.03
CF-3	2.1	8.6	1.69	2.06	0.64	2.06
CF-4	1.0	5.7	0.71	2.14	0.11	1.56
CF-5	0.5	5.9	0.90	2.06	0.13	1.90
CF-6	0.6	13.1	1.08	2.03	0.21	2.11
CF-7	1.8	8.1	0.86	2.03	0.18	1.95
CF-8	4.8	9.5	0.94	1.99	0.57	1.85
CF-9	4.6	12.4	0.95	1.99	0.38	2.03
CF-10	1.4	13.0	0.78	2.10	0.16	1.94
CF-11	4.8	16.8	0.81	1.87	0.42	2.17
Average	2.4	10.0	1.03	2.04	0.32	1.96

50 mg of old and symptomatic leaves of *Vitis vinifera* var. Cabernet Franc was used in RNA isolation using Spectrum™ Plant Total RNA Kit (Sigma) with standard protocol (Standard) or modified protocol with 2.5 % of PVP-40 being added in the lysis solution (Modified). The total RNA yield (μg), A260/A280 and A260/A230 ratios were determined with a NanoDrop spectrophotometer

### RNAs isolated using the modified method are suitable for the detection of both RNA and DNA viruses

*Grapevine red blotch-associated virus* (GRBaV) is a recently characterized DNA virus with a seemingly wide distribution in North American [6, 14, 52, 53]. Total nucleic acids or DNA extracts from grapevine tissues have been used for the detection of GRBaV [14, 53]. The results described earlier showed that the isolated total RNA using the modified Sigma system is of sufficient quality, and are suitable for RT-PCR to detect GRSPaV as a RNA virus (Fig. 2). It would be of great benefit if the total RNA extracts could also be used for the detection of the DNA virus - GRBaV. For this purpose, we compared the effectiveness of the total RNA extracts isolated with the modified method and DNA extracts isolated with Plant/Fungi DNA Isolation kit (Norgen BioTek) for detection of GRBaV. Both total RNA and DNA were isolated from cambium scraping of 10 individual vines and the same results were obtained for the detection of GRBaV by PCR using either RNA or DNA preparations as template (Additional file 3: Figure S2). The qPCR on the GRBaV-positive samples showed that the Cq for the RNA samples were 2–3 cycles lower than that for the DNA samples. This result demonstrated that the isolated total RNA using the modified method is also suitable for PCR to detect GRBaV. We have further shown that it was the genomic DNA of GRBaV and not the transcription products that served as templates for PCR amplification (data not shown). Thus, it can be concluded that the RNA isolation procedure is effective not only for RNAs but also for small DNA molecules as the genomic DNA of GRBaV.

### Leaf as well as cambium scrapings are reliable source for the detection of viruses in grapevine

The effectiveness of the modified method in isolating total RNA from old grapevine leaves collected in late fall was further validated by examining a large number of samples and both red and white wine varieties. These samples included several major wine varieties that are important for the cold climate viticulture and enology in Ontario: Cabernet Franc, Chardonnay, Riesling, Gamay and Gewurztraminer. To compare the effects of different types of tissue on yield and quality of isolated RNAs, we used both leaves and cambium scrapings as source materials for RNA isolation. The average yield of total RNA isolated from old leaves of 19 samples was 7.0 μg, higher than that from cambium scrapings (5.5 μg), with similar quality: 2.02 for A260/A280 and 1.98 for A260/A230 for RNAs from leaf tissues, and 2.07 for A260/A280 and 1.95 for A260/A230 for RNAs from cambium scrapings. RT-PCR was conducted to detect GRSPaV, GLRaV-2, GLRaV-3 and GRBaV using total RNA isolated from leaf and phloem samples (Table 2). Ubiquitin gene sequence, the internal reference control, was detected from all the samples tested, indicating the high quality of these RNA samples and the successful conditions of RT-PCR. The detection of GRSPaV and GLRaV-2 from leaf tissues is the same as that from cambium scrapings (Table 2). For GLRaV-3, consistent results were obtained for 17 of the 19 samples, with the exception of only two samples for which only RNAs isolated from leaf tissue produced a faint band after RT-PCR. For GRBaV, also 2 of 19 samples showed inconsistent results between leaf and phloem tissues (Table 2). This discrepancy may be related to its uneven distribution in these vines. Nevertheless, these results unequivocally demonstrate that both leaf tissue and cambium scrapings can serve as reliable source for the detection of several important grapevine viruses. RT-qPCR results support this conclusion, although in general a lower Cq value was obtained in qPCR using nucleic acids isolated from phloem scrapings for all three viruses tested (Table 2).

**Table 2:** RT-PCR and RT-qPCR detection of GRSPaV, GLRaV2, GLRaV3 and GRBaV from total RNA isolated from both leaf and cambium tissues of grapevines

Sample no.	GRSPaV		GLRaV-2		GLRaV-3		GRBaV		UBI	
	L	C	L	C	L	C	L	C	L	C
Syrah:										
SY-1	+	+	-	-	-	-	+	+	+	+
SY-2	+	+	-	-	+	+	-	-	+	+
SY-3	+	+	-	-	+?	-	+	+	+	+
SY-4	+	+	-	-	+?	-	+	+	+	+
SY-5	+	+	-	-	+	+	+	+	+	+
	32.3	28.1			18.3	15.2	19.0	14.7	25.2	22.0
SY-6	+	+	-	-	+	+	-	-	+	+
SY-7	+	+	-	-	-	-	+	+	+	+
	26.2	25.7			37.7	35.5	18.8	14.3	22.0	21.2
Gamay:										
GA-1	+	+	-	-	-	-	-	-	+	+
GA-2	+	+	-	-	-	-	-	-	+	+
Cabernet Franc:										
CF-1	+	+	-	-	-	-	-	-	+	+
CF-2	+	+	-	-	+	+	-	-	+	+
	30.4	28.4			17.1	4.5	ND	34.5	22.0	23.1
CF-3	+	+	-	-	-	-	-	-	+	+
Chardonnay:										
CH-1	+	+	+	+	+	+	-	+?	+	+
	26.2	27.4			17.1	14.5	ND	32.8	22.0	22.6
CH-2	+	+	-	-	+	+	-	-	+	+
CH-3	-	-	-	-	+	+	+	+	+	+
	ND	ND			20.9	16.9	19.4	15.0	28.1	23.6
Gewurztraminer:										
GE-1	-	-	-	-	+	+	-	-	+	+
GE-2	+	+	-	-	+	+	+?	-	+	+
Riesling:										
RI-1	+	+	-	-	+	+	-	-	+	+
RI-2	+	+	-	-	+	+	-	-	+	+

Nineteen grapevine samples randomly selected from six varieties of *Vitis vinifera* were used to isolate total RNA from leaf or cambium tissue with the modified Sigma kit. + and -: positive and negative, respectively, in the detection of viruses by RT-PCR. The primers used were listed in Additional file 1: Table S1 with RSP35 and RSP36 being used for GRSPaV. Cq of RT-qPCR was given for select samples where qPCR was conducted. ?: weak DNA band after PCR amplification. ND: not determined

## Discussion

Woody perennials constitute a group of economically important plant species with wide uses such as fruit crops, ornamentals, in urban landscaping and timber. Unfortunately, understanding of gene expression, regulation as well as many other biological processes of woody plants lags far behind those of herbaceous species. Perhaps due to the perennial nature of woody plants and the practice of vegetative propagation, woody perennials are hosts to large numbers of viruses and viroids [2, 4], which collectively represent a major roadblock impeding the productivity and sustainability of woody perennial crops. However, the biology and pathological properties of these woody plant viruses are poorly understood. This situation can be attributed to several factors that are inherent to woody plants, including the long time required for them to grow and the tremendous technical difficulties related to the isolation of sufficient quantities of pure nucleic acids. Challenges with nucleic acid isolation are mainly due to the fact that woody perennials generally contain high levels of secondary metabolites, such as polyphenolic compounds and polysaccharides [26, 54–58]. The presence of these secondary metabolites, especially phenolic derivatives, interferes with and even impedes the isolation of pure nucleic acids. Due to these reasons, studies of viruses infecting woody perennials have also fallen far behind compared to those infecting herbaceous plants. Clearly, it is imperative to develop and refine methodologies with which large amounts of pure nucleic acids can be readily isolated from woody perennials.

In this work, we first compared the effectiveness of five most commonly used commercial kits for the isolation of total RNA from two of the most important fruit crops, grapevine and peach. We have shown that the performance of these kits was highly plant-specific. We further simplified the procedure based on the Sigma and Norgen kits to render this technology suitable for high-throughput assays. We demonstrate that omission of liquid nitrogen, as well as mortar and pestle not only did not negatively impact on RNA isolation but, surprisingly, improved RNA yield without jeopardizing its quality. This modification is significant, as it would negate the requirement for liquid nitrogen, mortars and pestles, and their washing and sterilization before each use. In turn, this makes it possible that our modified system could reach a broader utilization by many potential users. Moreover, we modified the RNA isolation system by including PVP into the lysis buffer, which is shown to be highly effective for isolating highly pure RNA from both young and very old grapevine leaves collected throughout the entire growing season. The resulting RNAs were shown to be of sufficient quality as judged by RT-PCR, RT-qPCR and next generation sequencing (to be reported separately) for the detection of several major viruses that infect grapevine. Lastly, we have shown that the same isolation system was effective not only in isolating RNA but also small DNA molecules as judged by successful amplification of GRBaV, a recently discovered DNA virus from grapevines showing red blotch symptoms.

Interestingly, the performance of these five commercial kits is largely plant-specific. For example, when peach leaves were used, four of the systems (with the exception of the Bioneer kit) worked well in generating large quantities of total RNA of satisfactory purity (Fig. 1a). In sharp contrast, when grapevine leaves were used, only three kits (Sigma, Norgen and Bioneer) were effective. The Qiagen kit failed entirely (Fig. 1b), regardless of whether RLT or RLC was used as the lysis buffer. In line with our findings, Le Provost et al. [50] also reported that the Qiagen plant extraction system produced very low yields of RNA from leaves of pine, eucalyptus, oak and chestnut. On the other hand, the pellets containing RNAs isolated with TRIzol were brown in color, difficult to dissolve, and did not migrate into gel during electrophoresis (Fig. 1b). TRIzol also failed to isolate RNA from other plants, such as loquat (*Eriobotryya japonica*) [59], strawberry [49], among others. This was likely due to the fact that RNAs formed complexes with polyphenolics and polysaccharides during the homogenization step, a phenomenon frequently encountered during nucleic acid isolation from woody perennials [26, 54–58, 60].

The challenges in isolation of RNA from grapevines is further demonstrated by the fact that both Spectrum Plant Total RNA kit and Plant/Fungi Total RNA kit failed in isolating quality total RNA from older and symptomatic grapevine leaves, although they worked very well for young and mature leaves collected early in the season. However, we have resolved this issue by modifying the Spectrum Plant Total RNA kit through addition of 2.5 % PVP-40 to the lysis buffer (Table 1); the improved RNA isolation technology provided high quality and quantity of total RNA suitable for RT-PCR, RT-qPCR (this study) and next generation sequencing (to be reported separately). This study further signifies the critical effect of PVP-40 in isolating total RNAs from recalcitrant plants that contain high levels of secondary metabolites. Soluble PVP as well as insoluble PVPP at various concentrations have been used in RNA extraction from numerous woody perennials, including grapevine [27, 28, 54, 55, 57, 58, 60, 61]. The reasons for the beneficial effects of PVP and PVPP are not fully understood. It was reported that phenolic substances abound in different tissues of woody perennials, with grapevine being the most notorious. One type of such phenolic compounds are proanthocyanidins which, when present in high concentrations, complex with RNAs, rendering them unavailable [56]. It is believed that PVP and PVPP would form large and insoluble complexes with reactive phenolic substances, preventing them from forming complexes with nucleic acids, and hence leading to the release of nucleic acids into the homogenate during extraction.

Using leaf as source material for virus detection has significant advantages as compared with other tissues, such as cambium. First, ample leaf materials are available throughout the growing seasons. Second, collection and processing of leaf tissue are much easier and more convenient than petiole and phloem scrapings. Third, sampling of leaves causes no damage to the vine as compared to sampling of cane cuttings from which cambium scrapings are obtained. Fourth, leaves from multiple canes can be collected from a single vine or from several vines, making test of composite samples possible. This advantage becomes more prominent, as several samples from different parts of a vine are usually collected in virus testing in consideration of the uneven distribution of viruses in the infected grapevines [62–64]. Furthermore, sampling of composite samples from several vines is often used in large-scale disease surveys. Finally, easy and less processing means less chance for cross contamination between samples during virus testing. Unfortunately, grapevine leaf tissues, especially when older and diseased, are problematic in obtaining high quality DNA and RNA [27, 53], as they accumulate high levels of secondary compounds [27, 40]. Therefore, cambium tissue has been commonly used in the diagnostics and genome sequencing of viruses in grapevines [6, 20–25, 45, 65, 66]. The present study has shown that, by modifying the Spectrum™ Plant Total RNA kit with addition of 2.5 % PVP-40 to the lysis buffer, satisfactory quality and quantity of RNAs were isolated from older and symptomatic leaf tissues collected in late summer and the fall (Table 1). We then compared total RNA isolated from leaf tissue and cambium tissue for virus detection and found that leaf tissue can serve as a reliable source for detection of several major viruses, namingly GRSPaV, GLRaV-2, GLRaV-3, and GRBaV throughout the entire growing season. This improvement makes it possible to readily detect major viruses in grapevine throughout the year.

Only RNA viruses had been reported to infect grapevines up till 2011. However, a DNA virus and two retro-like viruses have been recently identified in grapevine as a result of the application of deep sequencing technologies [6, 8, 9]. For example, a new geminivirus, designated as *Grapevine red blotch-associated virus,* has been identified in grapes with red blotch disease [6, 14, 52]. A new virus that belongs to the genus *Badnavirus* (family *Caulimoviridae*) was discovered in grapevine showing vein-clearing and decline syndromes [9]. More recently, another new badnavirus was found associated with the Roditis leaf discoloration disease [8]. It is likely that additional new DNA viruses will be identified through deep sequencing in grapevines with other diseases of currently unknown etiology. Now both DNA and RNA viruses need to be targeted in virus screening in vineyards and nursery materials. Usually DNA-specific systems are needed for the detection of viruses with DNA genomes whereas RNA-specific systems are required for the detection of RNA viruses. We now find that the nucleic acid extraction system we have developed is sufficient for the detection of both DNA and RNA viruses in grapevines. This, in turn, would result in significant savings in both the cost of reagents and time in viral diagnosis and discovery. Our RNA isolation technology has been successfully applied to a wide range of other woody perennials, such as apple, cherry, nectarine, horse chestnut, maple, birch, pine and spruce. Further work is needed to verify whether the extraction protocol we have established is also applicable to many other woody plants as well as herbaceous plants for the detection of viruses with either RNA or DNA genomes.

The genomes of several economically important woody plants (e.g., grapevine, apple, peach, sweet orange, poplar, eucalyptus, and Norway spruce) have been sequenced. The subsequent endeavors would be to determine, through gene profiling and comparative genomics/transcriptomics, the function of genes and genetic elements that regulate gene expressions in order to answer the question of what genes make a tree that is fundamentally different from herbaceous plants [51]. The improved RNA isolation procedure developed in this study would also provide an assurance for high-quality RNAs required for such studies.

## Conclusions

In closing, we have shown that both Sigma and Norgen kits were highly effective in isolating quality total RNA from grapevines and a wide range of other woody perennials using young leaf tissues. We have further demonstrated that this RNA isolation procedure can be substantially simplified through the use of disposable BIOREBA bags during sample homoge nization, without the need for mortar and pestle and liquid nitrogen as recommended by vendors. Further modification of the system by including PVP-40 in the lysis buffer enabled the successful use of our method on both young and old leaves collected from the field throughout the entirety of the growing season. This improved nucleic acid isolation method would have wide applications in the diagnostics and discovery of viruses and viroids, studies on gene expression and regulation, transcriptomics, and small RNA biology in woody plant species.

## Methods

### Plant materials

Twelve species of diverse woody plants were included in this study. These plants included fruit crops (grapevine, peach, apple, cherry, nectarine and strawberry), ornamentals (lilac and horse chestnut) and trees (paper birch, Norway maple, white spruce and white pine). Grapevine leaves were collected from three varieties of *Vitis vinifera* (Chardonnay, Riesling, and Thompson seedless), *V. riparia* (from Quebec and Manitoba) from growth chamber in the University of Guelph. Grapevine leaves and canes were also collected from 6 varieties of *V. vinifera* (Chardonnay, Riesling, Syrah, Cabernet Franc, Gamay, and Gewurztraminer) from early summer (June) to late fall (November) from commercial vineyards in Niagara, Ontario. Leaves from paper birch (*Betula paperifera*), Norway maple (*Acer platanoides*), white pine (*Pinus strobus* L.), white spruce (*Picea glauca*) and horse chestnut (*Aesculus hippocastanum*) were collected in May from University of Guelph Arboretum. Leaves of peach (*Prunus persica*), lilac (*Syringa vulgaris*), strawberry (*Fragaria* × *ananass*), cherry (*Prunus avium*), nectarine (*Prunus persica*) and apple (*Malus domestica*) were collected in June from local gardens.

### Commercial RNA isolation kits

Five commercial kits were selected for this study, which included TRIzol Reagent (Life Technologies), RNeasy Plant mini kit (Qiagen), Spectrum™ Plant Total RNA kit (Sigma), AccuPrep viral RNA extraction kit (Bioneer) and Plant/fungi total RNA kit (Norgen BioTek).

### Methods of sample homogenization and RNA isolation

#### Sample homogenization

Three methods of sample homogenization were investigated.

**Method A:** This was the default method and used in initial experiments for all kits according to the manufacturers’ instructions. Plant leaf tissues were ground in liquid nitrogen to fine powder using a mortar and pestle and 50 mg of plant leaf fine powder was then transferred into a 1.5 or 2 ml microfuge tube and processed following instructions of the individual kit.

**Method B**: Leaf samples were homogenized in an extraction bag with a hand-held homogenizer, both purchased from BIOREABA (AG, Switzerland). One hundred milligrams of leaf tissue was ground in an extraction bag containing 1 ml of lysis solution with a hand homogenizer until the tissue was completely macerated. The bag was rolled up, placed in a 50 ml plastic centrifugation tube and spun down for 10 s in a swing bucket rotor at 4,000 rpm to collect the liquid. The lysate of about 500 μl (therefore similar amount of tissues, 50 mg, as in Method A was used) was then transferred into a 1.5 ml microfuge tube and processed following instructions of the individual kit.

**Method C**: Leaf samples were homogenized with mortar and pestle without liquid nitrogen. 100 mg of plant leaf tissue was ground in a mortar containing 1 ml of lysis solution with a pestle until the tissue was completely macerated. The lysate of 500 μl (therefore similar amount of tissues, 50 mg, as in method A was used) was then transferred into a 1.5 ml microfuge tube and from then on processed following protocol provided by the individual kit for RNA isolation.

The protocols of the five kits are briefly described as follows for the convenience of readers. More information about each kit can be found on the manufacturer’s instructions.

### TRIzol reagent (Life Technologies)

One milliliter of TRIzol Reagent was added to a 2 ml microfuge tube with 50 mg of plant tissue fine powder. The tube was vortexed vigorously, and then incubated at room temperature for 5 min. Two hundred microliter of chloroform was added and vortexed vigorously, followed by incubation at room temperature for 3 min. The sample was centrifuged at 13, 000 rpm for 15 min at 4 °C. The upper aqueous phase was collected into a new tube and 0.5 ml of 100 % isopropanol was added to the tube. The mixture then was incubated at room temperature for 10 min and centrifuged at 13,000 rpm for 10 min at 4 °C. The supernatant was discarded and the RNA pellet was washed by applying 1 ml of 75 % ethanol, vortexing briefly and centrifuging the tube at 10,000 rpm for 5 min at 4 °C. The wash was discarded and the RNA pellet was air-dried for 5–10 min. The RNA pellet was then resuspended in 50 μl of RNase-free water by pipetting the solution up and down several times, followed by incubation at 55–60 °C for 5–15 min in a water bath.

### RNeasy plant mini kit (Qiagen)

450 μl of buffer RLT or RLC was added to a 1.5-ml microfuge tube containing 50 mg of plant tissue fine powder. The tube was vortexed vigorously and incubated at 56 °C for 3 min. The lysate was transferred to a QIAshredder spin column placed in a 2 ml collection tube, and centrifuged for 2 min at 12,000 rpm. The supernatant was then transferred to a new microfuge tube and 0.5 volume of ethanol (96–100 %) was added and mixed immediately by pipetting. The sample (usually 650 μl) was then transferred to an RNeasy spin column placed in a 2-ml collection tube and centrifuged for 15 s at 10,000 rpm. The flow-through was discarded. The RNeasy spin column was washed once by applying 700 μl buffer RW1 to the column, centrifuging for 15 s at 10,000 rpm and then discarding the flow-through. The column was then washed twice by applying 500 μl buffer RPE to the column, centrifuging for 15 s at 10,000 rpm, followed by discarding the flow-through. The column was centrifuged at full speed for 1 min to thoroughly remove the wash buffer. For RNA elution the RNeasy spin column was placed in a new 1.5 ml tube and 50 μl RNase-free water was added directly to the spin column membrane. The column was centrifuge for 1 min at 10,000 rpm to elute the RNA.

### Spectrum™ plant total RNA kit (Sigma)

500 μl of lysis solution with β-mercaptoethanol were added to a 2-ml microfuge tube containing 50 mg of plant tissue fine powder. The tube was vortexed vigorously and incubated at 56 °C for 3 min. The lysate was centrifuged at 14,000 rpm for 3 min and then the supernatant was transferred into a Filtration Column seated in a 2 ml collection tube, and centrifuged for 1 min at 14,000 rpm. The clarified flow-through lysate was then transferred to a new microfuge tube and 250 μl of Binding Solution was added and mixed immediately by vortexing. The mixture was applied to a Binding Column seated in a 2-ml collection tube and centrifuged for 1 min at 14,000 rpm. After discarding the flow-through, the column was washed once by applying 500 μl Wash Solution 1 to the column, centrifuging for 1 min at 14,000 rpm and then discarding the flow-through. Afterwards, the column was washed twice by applying 500 μl Wash Solution 2 to the column, centrifuging for 1 min at 14,000 rpm and then discarding the flow-through. The column was centrifuged at full speed for 1 min to thoroughly remove the wash buffer. Finally, the spin column was placed in a new 2-ml Collection Tube and 50 μl Elution Solution was applied to the column. The column was then centrifuged for 1 min at 14,000 rpm to elute the RNA.

### AccuPrep viral RNA extraction kit (Bioneer)

To a 1.5 ml microfuge tube with 50 mg of plant tissue fine powder, 400 μl of Binding Buffer was added and mixed by lightly vortexing. After incubation for 10 min at room temperature, 100 μl of isopropanol was added and lightly vortexed for about 5 s, and then spun down for 1 min. The liquid was transferred into the binding column and centrifuged for 1 min at 8,000 rpm. Following centrifugation, the binding column was transferred to a new 2-ml collection tube and the column was washed by adding 500 μl of W1 buffer to the column, and centrifuging for 1 min at 8,000 rpm and then washed again by adding 500 μl of W2 buffer, and centrifuging for 1 min at 8,000 rpm. The column was spun down once more at 13,000 rpm for 1 min to completely remove W2 buffer. The binding column was transferred to a 1.5 ml collection tube, and 50 μl of Elution Buffer (pre-warmed to 60 °C) was added to the column. After standing for 1 min, the column was centrifuged at 8,000 rpm for 1 min to elute the RNA.

### Plant/fungi total RNA kit (Norgen BioTek)

600 μl Lysis Buffer C was added to a 2-ml centrifuge tube containing 50 mg of plant tissue fine powder. The tube was vortexed vigorously and incubated at 56 °C for 5 min. The lysate was then transferred to a filter column placed in a 2-ml collection tube, and centrifuged for 2 min at 14,000 rpm. The supernatant was then transferred to a new microfuge tube and an equal volume of ethanol (96-100 %) was added and mixed immediately by votexing. 600 μl of the clarified lysate with ethanol was applied onto a spin column placed in a 2-ml collection tube and centrifuged for 1 min 14,000 rpm. After discarding the flow through, the column was resembled. The remaining lysate was then loaded onto the column and re-centrifuged for 1 min at 14,000 rpm. The column was washed three times by applying 400 μl of Wash Solution A to the column, centrifuging for 1 min and then discarding the flow-through. The column was centrifuged for 2 min to thoroughly dry the resin. Finally, the spin column was placed in a new 1.5 ml tube and 50 μl Elution Solution A was applied to the column. The column was then centrifuged for 2 min at 200 rpm, followed by 1 min spin at 14,000 rpm to elute the RNA.

### Assessment of yield and quality of total RNA and small RNA

The purity and concentration of the total RNA preparations were assessed with a NanoDrop spectrophotometer (ND-1000, Thermo Scientific, Delaware, USA) at the wavelengths of 230, 260, and 280 nm. RNA integrity was verified based on the 28S and 18S rRNA bands after electrophoresis on 1.5 % formaldehyde–agarose gel, stained with ethidium bromide and visualization with UV light. The integrity and size distribution of purified RNAs were also evaluated with a 2100 Bioanalyzer (Agilent Technologies, Inc. Santa Clara, CA) equipped with an RNA Nano chip. Small RNAs and miRNAs from the total RNA samples isolated from young grapevine leaves with the five commercial kits were analyzed by the Agilent Bioanalyzer equipped with Small RNA Analysis kit (Agilent Technologies, Inc.) and the yields of small RNAs and miRNAs were calculated using the software Bio sizing version B.02.08. SI648 (SR2).

### RT-PCR and RT-qPCR

Total RNA from grape leaves isolated with kits from Sigma, Bioneer and Norgen were used in RT-PCR and RT-qPCR. Synthesis of cDNA was performed using the High-capacity cDNA Reverse Transcription kit (Life Technologies). The reaction mix (20 μl) included 500 ng of total RNA, 2.0 μl 10X RT Buffer, 0.8 μl 25X dNTP Mix (100 mM), 2.0 μl 10X RT Random Primers, 1.0 μl Multiscribe™ Reverse Transcriptase (50U/μl). The mix was incubated for 10 min at 25 °C, then 120 min at 37 °C, followed by incubation for 5 min at 85 °C, and then stored at 4 °C for immediate use or −20 °C for later use. PCRs were performed on a PTC-2000 Peltier Thermal Cycler (MJ Research). 25 μl of PCR reactions contained 2.5 μl of 10X PCR buffer, 0.5 μl of dNTPs (10 mM), 0.5 μl of forward primer and reverse primer (10 μM each) (see Additional file 1: Table S1 for the primers used), 0.5 μl of Taq polymerase and 1 μl of cDNA. PCR conditions included an initial denaturation step at 94 °C for 4 min, then 35 cycles at 94 °C for 30 s, 50 °C for 30 s and 72 °C for 1 min, followed by a final extension at 72 °C for 7 min. The PCR products were analyzed on 1.5 % agarose gel, followed by staining with ethidium bromide.

qPCRs were performed on StepOnePlus (Applied Biosystems) in the Genomics Facility of University of Guelph. 15 μl of reaction mix contained 7.5 μl of *Power* SYBR® Green PCR Master Mix (Applied Biosystems), 0.6 μl of F/R primer mix (5 μM each), 5 μl of cDNA diluted 5X in water and 1.9 μl of water. Cycling conditions included an initial denaturation at 95 °C for 10 min, then 40 cycles at 95 °C for 15 s, 55 °C for 30 s and 72 °C for 30 s, followed by melting curve analysis to confirm the specificity of PCR amplification. The primers and RT-qPCR conditions for miRNA 156a and 159a were described by Wu et al. [67]. The quantification cycle (C_q_) values were calculated using the threshold cycle method [47].

## Additional files

Additional file 1: Table S1. Primers used in this study for the detection of viruses and plant internal reference genes. **Table S2.** Small RNA and miRNA isolated from grapevine leaves. (PDF 104 kb) Additional file 2: Figure S1. RT-PCR detection of GRSPaV and ubiquitin gene using RNAs isolated from old grapevine leaves with Spectrum™ Plant Total RNA kit (Sigma) with standard method (**A** and **C**) or modified method (**B** and **D**). (**A**) and (**B**): Agarose gel analysis of RT-PCR products amplified with primers RSP35 and RSP36 (Additional file 1: Table S1) on 11 total RNA extracts listed in Table 1 with Method A or B respectively. (**C**) and (**D**): Agarose gel analysis of RT-PCR products amplified with UBI primers on these 11 total RNA extracts using Method A and Method B respectively. M: molecular size marker (bp); lane 12: water. (PPT 1035 kb) Additional file 3: Figure S2. qPCR detection of GRBaV from nucleic acid preparations isolated using commercial kits as recommended for RNA and DNA. (**A**) Agarose gel analysis of PCR with GRBaV primers (Additional file 1: Table S1) on extracts from 10 vines purified with Plant/Fungi DNA Isolation kit. (**B**) Agarose gel analysis of PCR products on total extracts from the same 10 vines isolated with modified Sigma system for plant total RNA. M: molecular size marker (bp); lane 11: water. (PPT 493 kb)
