# Supplementary material for: A highly effective and versatile technology for the isolation of RNAs from grapevines and other woody perennials for use in virus diagnostics
Source: Virol J. 2015 Oct 20;12:171. doi: 10.1186/s12985-015-0376-3 (PMC4615883; doi:10.1186/s12985-015-0376-3)
Supplement: Additional file 1: Table S1. — Primers used in this study for the detection of viruses and plant internal reference genes. Table S2. Small RNA and miRNA isolated from grapevine leaves. (PDF 104 kb) [file 12985_2015_376_MOESM1_ESM.pdf]

Table S1. Primers used in this study for the detection of viruses and plant internal reference genes.

Target	Primer	Primer sequence (5’-3’)	Amplicon size (bp)	Gene
**GRSPaV**	RSP21	GAGGATTATAGAGAATGCAC	441	Coat protein
	RSP22	GCACTCTCATCTGTACTCC		
	RSP35	AGRYTTAGRGTRGCTAARGC	476	RNA-dependent RNA polymerase
	RSP36	CACATRTCATCVCCYGCAAA		
	RSP8277F	AACTGGGCCAAGAAAGGATT	160	Coat protein
	RSP8436R	CTTGAACCTCAAAAGAGGCG		
**GLRaV-2**[a]	LR2-9239F	ATGGTGAARCGYGAYGCTAA	292	RNA-dependent RNA polymerase
	LR2-9530R	TCACGAAWGCRTCYTGAGAC		
**GLRaV-3**[b]	LR3-6995F	GGGRACGGARAAGTGTTACC	144	RNA-dependent RNA polymerase
	LR3-7138R	TCCAAYTGGGTCATRCACAA		
**GRBaV**	GRBaV1097F	ACGAGGAATCGTTTGAATCG	235	Coat protein
	GRBaV1331R	TAAACGTATGTCCACTTGCAG		
**Ubiquitin**[c]	UBI-F	CCGCACTCTTGCTGATTACA	146	Internal reference control
	UBI-R	GTGCATAACATTTGCGGCAG		
**Actin 1**[d]	ACT1-F	TGCTGACAGAATGAGCAAGG	147	Internal reference control
	ACT1-R	GAGATCCACATCTGCTGGAAG		

[a] Primers used for the detection of GLRaV-2 were from Alabi et al. [45]. [b] Primers were from Bester et al. [46]. [c] *V. vinifera* gene encoding ubiquitin-60S ribosomal protein L40-2 (UBI) (GenBank accession no. XM_002273532). [d] *V. vinifera* actin 1 (ACT1) (GenBank accession no. XM_002282480).

Table S2. Small RNA and miRNA isolated from grapevine leaves.

**Kit**	**Small RNA (µg)**	**miRNA (ng)**	**miR 156a (C_q)**	**miR 159a (C_q)**
**Sigma**	0.01	1.67	27.60	21.41
**Norgen**	4.15	142.93	22.61	13.86
**Bioneer**	1.15	47.95	24.70	17.40
**Qiagen**	0.04	1.28	26.26	19.91
**TRIzol®**	0.01	3.92	N/A	N/A
**NTC**			N/A	35.87

The yields of small RNA and miRNA from the RNA samples isolated from grape young leaves with the five commercial kits were determined by Agilent 2100 Bioanalyzer analysis (also see Fig. 1C) and calculated using the software Bio sizing version B.02.08. SI648 (SR2). The miRNAs 156a and 159a were detected by RT-qPCR with specific primers to each target. The C_q values were calculated using the threshold cycle method [47]. N/A: no amplification due to the presence of inhibitors; NTC: no template control.
