# Supplementary figures and images for: A highly effective and versatile technology for the isolation of RNAs from grapevines and other woody perennials for use in virus diagnostics
Source: Virol J. 2015 Oct 20;12:171. doi: 10.1186/s12985-015-0376-3 (PMC4615883; doi:10.1186/s12985-015-0376-3)

## Slide 1
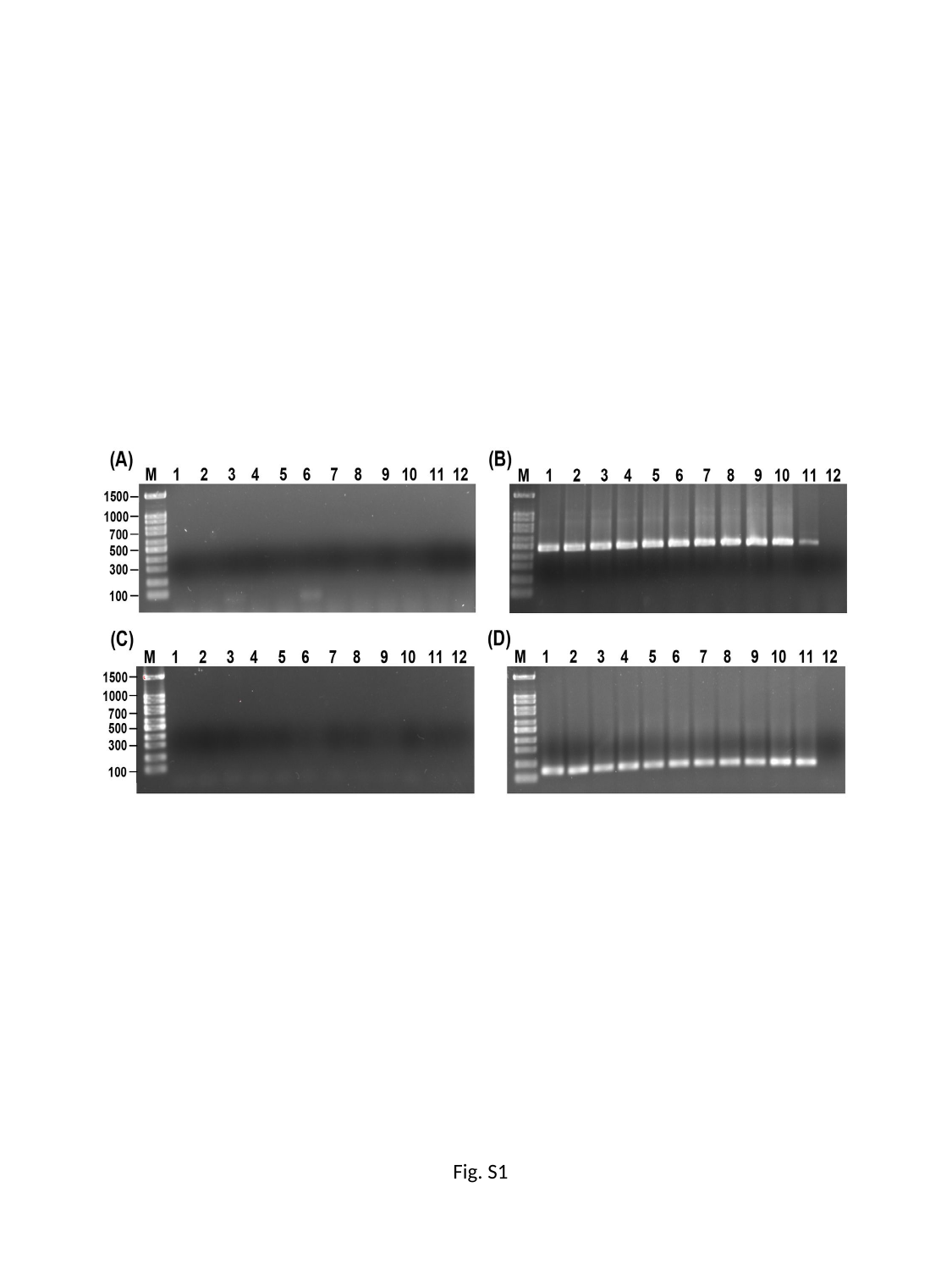

Fig. S1

Supplement: Additional file 2: Figure S1. — RT-PCR detection of GRSPaV and ubiquitin gene using RNAs isolated from old grapevine leaves with Spectrum™ Plant Total RNA kit (Sigma) with standard method (A and C) or modified method (B and D). (A) and (B): Agarose gel analysis of RT-PCR products amplified with primers RSP35 and RSP36 (Additional file 1: Table S1) on 11 total RNA extracts listed in Table 1 with Method A or B respectively. (C) and (D): Agarose gel analysis of RT-PCR products amplified with UBI primers on these 11 total RNA extracts using Method A and Method B respectively. M: molecular size marker (bp); lane 12: water. (PPT 1035 kb) [file 12985_2015_376_MOESM2_ESM.ppt]

## Slide 1
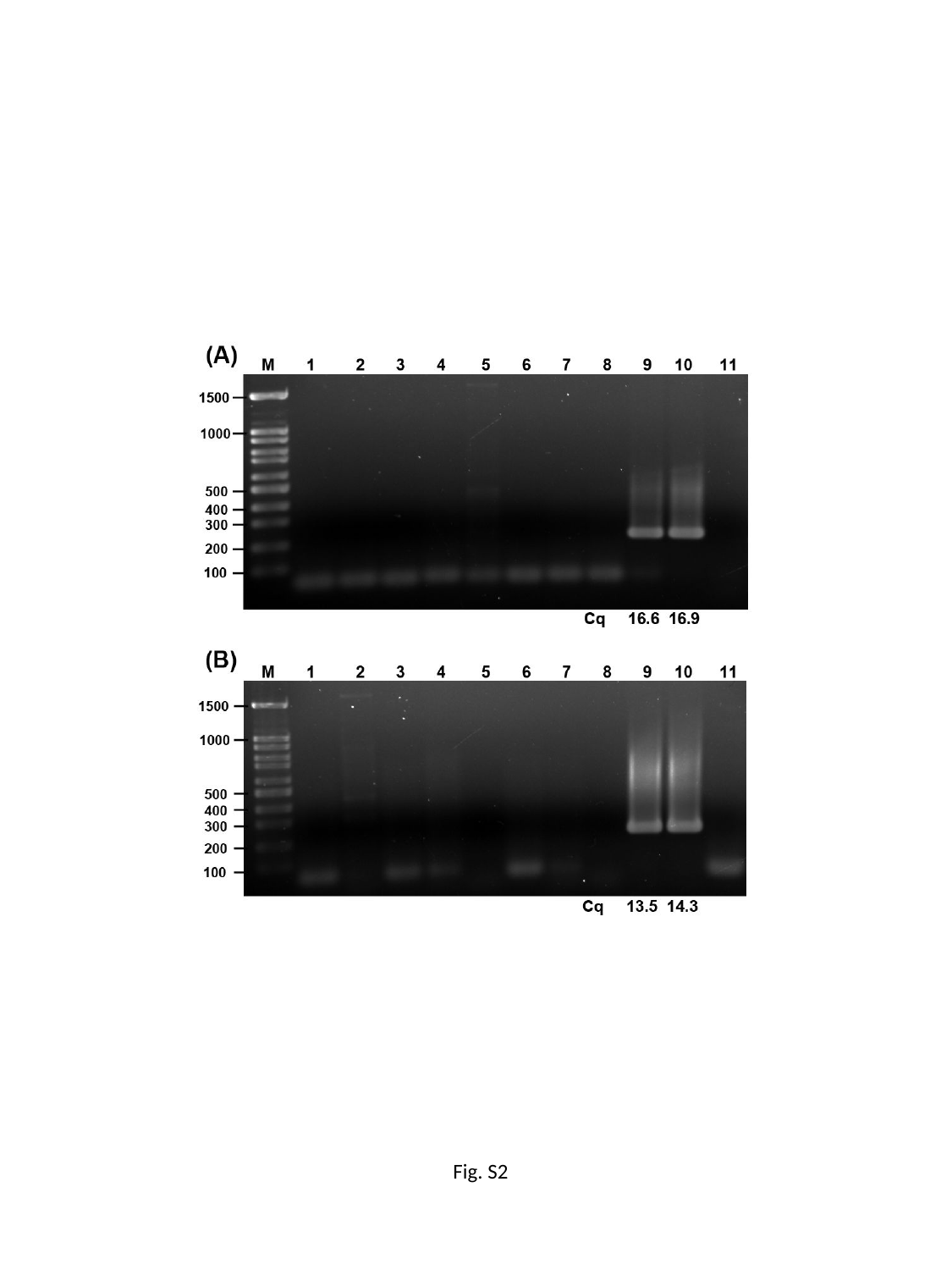

Fig. S2

Supplement: Additional file 3: Figure S2. — qPCR detection of GRBaV from nucleic acid preparations isolated using commercial kits as recommended for RNA and DNA. (A) Agarose gel analysis of PCR with GRBaV primers (Additional file 1: Table S1) on extracts from 10 vines purified with Plant/Fungi DNA Isolation kit. (B) Agarose gel analysis of PCR products on total extracts from the same 10 vines isolated with modified Sigma system for plant total RNA. M: molecular size marker (bp); lane 11: water. (PPT 493 kb) [file 12985_2015_376_MOESM3_ESM.ppt]
